# Medical and Physical *Intelligence*

**Published:** 1799-03

**Authors:** 


### MEDICAL AND PHYSICAL

### *INTELLIGENCE,*

### (Original and Selected.)

THE *yellow fever*, whofe ravages within a few years paft have been fo often deplored, has lately renewed its baneful vifitation in the United States, to a greater extent than hitherto remembered, and with multiplied horrors. The northern, fouthern, and weftern parts of the Union have for the moft part been fo fortunate as to efcape this calamity. The commercial towns fituated to the eaftward of the bay of Chefapeak, and the river Sufquehanna, have been the principal fcene of fuffering. Portfmouth, in New Hampfhire, Bofton, New London in Connecticut, New York, Philadelphia, and Wilmington, in the ftate of Delaware, befides fome other places of inferior confideration, have largely fmarted under this epidemic fcourge. Portfmouth and Peterfburgh in Virginia, were the only places weft and fouth of the Chefapeak and Sufquehanna, fo far as we yet know, where the fever raged with violence. In all thefe towns the ficknefs and mortality have been confiderable; and, in addition to thefe, the confternation and flight of the inhabitants, and the derangement and fufpenfion of bufinefs, with all the concomitant evils and inconveniencies, have greatly contributed to increafe the public diftrefs.

Although the months of June and July, with the exception of a few days, had been moderate, cool, and rainy, the weather totally changed in Auguft, when it exhibited fuch a degree of heat, in point of duration and fteadinefs, if not of feverity, as is very feldom witneffed in that climate. Throughout a great part of September, the heat continued with little fign of abatement; even much of October was unfeafonably warm; and the froft did not properly fet in till a later period of the feafon than ufual.

This epidemic generally appeared early in Auguft; through the remainder of which month, as well as September, and a great part of October, it continued to rage with undiminifhed violence: it was not until the latter end of October, although in fome inftances earlier, that a fenfible abatement in the number of its victims had taken place, and in the firft weeks of November, the diforder entirely ceafed. During this fhort interval thoufands of ufeful citizens were prematurely cut off, among whom we have to deplore the lofs of not lefs than fixteen phyficians, and particularly that of the learned and univerfally refpected Dr. E. H. Smith, of New York!

---

It is with great fatisfaction that we can at length announce the difcovery of a *more fuccefsful method of treating the Yellow Fever*, in America, than any which has been hitherto attempted. We learn from the report of Meffrs. Ifaac Rand and John Warren, relative to the anatomical diffection of bodies dead of the late malignant Epidemic at Bofton, which report is figned September 8th, 1798, that, after proper evacuations, the ufe of Calomel has been found generally fufficient to anfwer the above important purpofe. This medicine medicine has been accordingly adminiftered to fifteen patients, within eighteen days; all of whom, excepting one, have either recovered, or have paft the dangerous crifis of the diforder. It has been given, not in the ufual dofes, for the purpofe of an evacuant by the inteftines, but in fmall dofes of one, two, or three grains every hour or two, fo as to produce a falivation as foon as poffible; with this view from 100 to 230 grains of calomel have been taken by the patient, in the courfe of three or four days, commencing the ufe of it immediately after the firft copious evacuations by bleeding and purging; and, in every inftance, as the falivation came on, the difeafe has been obferved to abate.

As coinciding in fentiment refpecting the ufe of mercury, fo as to produce a falivation, (in the treatment of the yellow fever) we with pleafure mention the learned Dr. Rufh of Philadelphia. But this method is ftill more explicitely pointed out, and more highly recommended by James Clark, M. D. F. R. S. E. in a treatife on the yellow fever, on the appearance of that contagious malady, in the ifland of Dominica, in the years 1790, 94, 95, and 96. The Doctor recommends the free ufe of mercury, both as a remedy and a preventative—His words are, "The officers of the army and navy, who have leifure and can be prevailed upon, on their arrival in the Weft Indies, to undergo one or two courfes of mercury, take a few laxative medicines, after confining themfelves to a moderate ufe of wine, and living chiefly on vegetables and fruits for the firft two months after their arrival, may rely, almoft to a certainty, on efcaping the fever."

---

In a *Meteorological Journal*, accompanied with remarks on the difeafes of the feafon and their treatment, publifhed by that accurate obferver, Dr. Shadrick Ricketfon, of Dutchefs County, we find the following, among other curious and interefting obfervations, on the three fpring-months, towards the clofe. "It may not be wholly foreign or improper to remark, that *horfes* were very generally and feverely afflicted this fpring (1797) with the common diftemper in the glands of the head and throat; for it has frequently been obferved that horfes and dogs have been affected as concomitant, or prefaging, epedimic or peftilential difeafes; but, whether the prefent or a future ficknefs is to follow, I leave for future obfervation to determine."—In concluding his remarks on the weather and difeafes of the laft fummer, the Doctor fays. "In my laft I mentioned that horfes were feverely afflicted with their common diforder, which has continued to be the cafe, but not fo generally; and I may now add that *dogs* have been in like manner violently difordered with a cough, &c. during the greateft part of the fummer."

---

Mr. Webfter's *Hiftory of Peftilential Difeafes*, particularly with a view to afcertain and defcribe thofe of America, is now in the prefs at New York.—The defign of this publication is, to collect the principal facts which regard the origin, progrefs, and declenfion of peftilential epedemics, in general; together with the remarkable phenomena of the natural world, fuch as unufual feafons, contagious difeafes among animals, uncommon appearance of infects, and other preceding or concomitant events which may probably have fome connexion with the latent caufes of peftilence; in order to obtain, if poffible, fome well-grounded knowledge of the fources from which proceed the wide-wafting difeafes which afflict mankind. A great number of curious facts are combined, and it is believed they will throw much light on this melancholy fubject, of fuch important concern to the commerce of our cities, and to the lives and happinefs of mankind.

It

We obſerve, with no ſmall ſatisfaction, the re-publication, in Philadelphia and Boſton, of the *Eſſays* of the philanthropic American, Count Rumford; a work which merits a place in the libraries, and even in the hands of all who ſincerely wiſh to promote the proſperity of their country, and the happineſs and proſperity of their fellow-mortals.

---

A ſecond volume of Dr. Trotter's *Medicina Nautica* is now in the preſs, and will be publiſhed early in March. This volume, we underſtand, contains an extenſive correſpondence with naval ſurgeons in different parts of the world. The diſcuſſion of the extirpation of contagion is reſumed, and much freſh evidence is adduced of the inefficacy of the nitrous gas, and all ſpecies of fumigation in this proceſs. A ſummary of Profeſſor Mitchill's doctrine on *Peſtilential Fluids* is given in an Appendix, ſelected from American Papers, tranſmitted to the Board of Admiralty by his Majeſty's Conſul General.

This doctrine directly oppoſes that of Dr. J. C. Smyth, and agrees in a great part with the practice of the naval phyſician, whoſe ſucceſs in the ſhips of the fleet, it muſt be confeſſed, has been ſuch as to make infection no longer formidable.

---

Mr. G. Cornelli, of Cologne, ſome time ago publiſhed a treatiſe (in German), *On the Diſadvantages attending the common Proceſs of Diſtilling Vinegar, &c.* in which, among other ſubjects, he endeavours to prove, that vinegar, when ſuddenly evaporated on hot bricks, or iron, for the purpoſe of fumigating ſick and bed-rooms, inſtead of purifying the corrupted air in ſuch places, has rather a contrary tendency. Hence by the uſual method of evaporating vinegar, the air becomes tainted with a great proportion of carbonic acid gas, one of the moſt irreſpirable ſpecies of air. The author, therefore, adviſes to place the vinegar, in ſome ſhallow veſſel, upon a moderately warm part of an oven or ſtove; but, as theſe are rarely uſed in Britain, the purpoſe may be fully anſwered, by placing a tin veſſel containing vinegar over a faint lamp, at ſuch a diſtance as to keep the vinegar near the boiling point, and thus communicating an equal degree of heat to the veſſel fixed above the lamp.

---

Since the death of the celebrated Deſſault, in 1795, M. Pelletan has been, and ſtill is, the principal ſurgeon at the *ci-devant Hotel Dieu*, now called *Hoſpice de l'Humanité*, at Paris. This ſtupendous aſylum for afflicted perſons of every deſcription, contained, previous to the Revolution, a ſufficient number of beds to accommodate at leaſt 2000 patients. And although this number is conſiderably leſs at preſent (a remark which equally applies to the population of Paris and the country of France at large); yet this reduction with reſpect to the hoſpitals, is attended with one moſt important advantage, that there is no neceſſity, at preſent, to crowd ſeveral individuals into one bed, or as a late traveller ſarcaſtically expreſſed it, "to pickle them together like herrings in a caſk."

The ſecond ſurgeon is Giraud, a worthy pupil of the late Deſault; in the ſix winter-months he delivers a regular courſe of lectures on anatomy and ſurgery, while Pelletan only makes occaſional diſcourſes and remarks on the clinical patients under his care. Giraud is an excellent and ſucceſsful operator, and is aided by a number of pupils, who partly reſide altogether in the hoſpital, and partly repair thither to viſit the patients, and to aſſiſt at the daily operations. The latter are called *Externes*, and the former, *Internes*, who keep a regular journal of the patients, as well as of the remedies preſcribed, and are ſtyled *Chirurgiens de Garde*, having each of them a ſeparate hall or ward of patients intrusted to their provisional care. Prior to the new order of things, the nuns waited on the patients in the character of nurses, and had, upon the whole, an almost unlimited sway in the house; latterly, however their influence has declined, or is in a manner lost, and they are now reduced to their original condition of waiters on patients, and are obliged to conform to the ordinary apparel of females. It should be observed, however, that the superintendance of the midwifery-wards in the house appertains to them exclusively; and they execute their trust in so strict a manner, that no male visitors whatever are admitted there, not even the *chirurgien en chef*, unless his assistance be essentially and expressly wanted, and called in by the nuns.

Notwithstanding the numberless chirurgical operations occurring here every day in the year, it is believed that the pupils or visitors derive no very great benefit from their attendance. The beds of the patients are placed too close together; there is no particular room appropriated to the operations, which, strange to tell, are performed on the very beds of the sufferers; the number of pupils and attendants present, together with the prodigious bustle, throng and noise—all these circumstances conspire to lessen the benefit which a visitant, particularly a foreigner, might otherwise receive in attending the Grand Hotel Dieu.

---

Another clinical establishment in Paris, of less magnitude, but much greater practical utility to the student, and infinitely better adapted to the purposes of cleanliness, as well as the performance of chirurgical operations, is that which formerly was known by the name of *la Charité*, but now by that of *Hospice de l'Unité*. The wards of this hospital are not so crowded with patients; the beds stand at a convenient distance from each other, and the number of students is not so great as in the *Hospice de l'Humanité.* Generally, about one hundred chirurgical patients are admitted here. The principal surgeon of this hospital is *Deschamps*, who being advanced in years, allows *M. Boyer*, who is likewise appointed one of the principal surgeons to the house, and an eminent pupil of Dessault, to perform most of the operations. Deschamps had formerly much and successful practice in lithotomy. He has lately published a large treatise on this subject, and has also acquired considerable reputation by a newly-invented instrument, which is now employed with great advantage in the operation for the popliteal aneurism.—Boyer is universally allowed to be the most ingenious of Dessault's pupils; he is not only an excellent operator, but he is ever apt and ready to instruct others, who address themselves to him with confidence, without displaying his great superiority, or dispatching the questions of his pupils with categorical and evasive answers. His anatomical and chirurgical lectures, as well as his work lately published on these subjects, are intitled to much praise for correctness of language and perspicuity of demonstration.—Although he was, for several months, provisionally appointed first surgeon to the Hotel Dieu, after the mysterious death of Dessault, yet, notwithstanding his great merit, he could not obtain this situation as a permanent office: *Dii non sic voluere.*

---

It is a singular fact, that even in France, where surgery has been so superiorly cultivated for centuries past, we meet with surgeons who are in a manner strangers to the treatment of internal disorders, or who at least do not interfere in the management of any other but what are strictly called, surgical patients. If, therefore, it should happen that a violent fever supervene in any chirurgical cafe, or after an operation performed by a furgeon, a phyfician is neceffarily called in, who frequently prefcribes medicines according to the prefent febrile fymptoms, without paying due regard to the external condition of the patient.—Such diftinctions however, between two infeparable branches of the fame profeffion, can never be productive of falutary confequences, but rather muft have an obvious tendency to check the practical progrefs of both.

---

The "*Annales de Chimie*" of the 30th Frimaire, (December 20, 1798) contain an interefting Memoir read by FOURCROY in the medical fchool at Paris, "*On the application of pneumatic chemiftry to the practice of medicine, and on the medical properties of oxygenated fubftances.*"

Our limits will not permit us, in this Number, to give an abftract of this elaborate effay, and to do it that juftice which its merits, and the celebrity of its author demand. We fhall therefore confine ourfelves to extract what C. Fourcroy calls his "*profeffion de foi.*"

After an appropriate introduction, alluding to the practical advantages refulting from new chemical difcoveries, and the good effects to be expected from them in medicine, he thus proceeds: "But if I confidently announce the hope of a happy and approaching revolution in the art of healing, I ought, while I appear to encourage fuch a change, to refift the dangerous confequences of that petulant activity, which inflames inftead of enlightening the mind; of that premature love of innovation, which is eager to deftroy without being able to raife any ftructure on the fcattered ruins; I fear, I confefs, as much from thefe imprudent innovators, as I apprehend from the zeal of the adherents to old fyftems. If the latter fet their faces againft the progrefs of reafon, the former are anxious to precipitate themfelves into errors, by exaggerations not lefs dangerous. I am equally an enemy to the innovating folly of the one, and to the dull inactivity of the other. I reject the pretended fufficiency of the Brunonian doctrine for every medical theory, as well as the indifcreet attempt of explaining every where the mechanifm of animal life, by a chemical action. I undoubtedly am defirous of a revolution in the theory of medicine; it is the object of my prayers; I have foretold it thefe fifteen years in my lectures; I have every where proclaimed it in my works; I fhall forward its progrefs with all my power and abilities:—but I wifh for a revolution, wife, moderate, and reflecting. I do not burn the old books, with Paracelfus; I do not break the pneumatic vafes; I do not at once profcribe every fpecies of medical knowledge; I preferve all that exifts, and do not facrifice this knowledge to any new applications, or to a doctrine hitherto built upon fand. It would be injudicious to abandon what we have induftrioufly acquired; to extinguifh at once the light kindled by long experience; and to exchange it for that *theoretical empiricifm*, which directs the medical practitioner to embrace a phantom."

---

Dr. SCHERER whofe merits in chemical fcience have already been noticed, p. 69, of this Journal, has lately commenced a periodical work which, by his extenfive correfpondence with the moft celebrated European chemifts, appears to poffefs confiderable advantages over all publications of a fimilar nature. This work is intitled the *Univerfal Chemical Journal*, and is publifhed in numbers, every month, by Breitkopf and Hartel at Leipzig; it promifes to become one of the moft valuable vehicles of phyfical and chemical knowledge on the continent.—Dr. Scherer has alfo announced another publication he is preparing for the prefs, intitled "*Inquiries into the nature and application of every fpecies of manure hitherto known; inftituted by the members of the Board of Agriculture in London.*"

It is with great fatisfaction we can announce to our readers that the fourth and laft volume of the "*Hiftory of Medicine*" (a valuable work begun about feven years ago!) by the accurate and very learned profeffor KURT SPRENGEL, of Halle, will make its appearance in the month of May next; we may alfo indulge the hope of feeing fpeedily a correct tranflation of it by Doctor ANDREW DUNCAN Jun. of Edinburgh.—To afford the reader fome idea of this very refpectable work, alike interefting to the philofopher and the phyfician, we fhall only obferve, that in order to write an hiftory of medicine with advantage, it was neceffary to collect and bring under diftinct points of view, the facts and arguments fcattered in a thoufand works; to read the writers of different ages and nations, *in the original;* to enter into the fpirit of the times when they were compofed, and to inveftigate the hiftory of fociety and the fciences, in all the branches connected with medicine. This great and laborious undertaking Profeffor Sprengel has been able to accomplifh; nor has he ever tamely copied from his predeceffors, but with indefatigable zeal drawn his knowledge from the original fources.

---

Two eminent phyficians, Profeffor JUNCKER, of Halle, and Dr. FAUST, of Buckeburg in Germany, are now (and have been for feveral years paft) actively employed in circulating and preparing for its execution no lefs important a fcheme than that of fubduing, and, if poffible, extirpating the natural *fmall-pox*, as a contagious difeafe, throughout Europe; in a manner fimilar to that, by which the progrefs of the plague and leprofy had been fuccefsfully checked, for feveral centuries paft *. Many refpectable phyficians, both in Germany and Italy, are zealoufly co-operating in this humane and meritorious defign, particularly Dr. HUFELAND, of Jena, and Profeffor SPRENGEL, of Halle.

We are authorized to ftate that the Congrefs, affembled at Raftadt, have taken this fubject into ferious confideration, although it is in a manner unconnected with the important objects of their miffion.

Dr. Fauft has lately addreffed a printed circular letter to the different public agents convened there; and, much about the fame time, another memoir to the fame effect was tranfmitted to Raftadt by Profeffor Juncker, although without his previous knowledge of Dr. Fauft's fpirited addrefs, which was introduced by a gentleman refiding there in a diplomatic capacity, and is printed in the German and French languages.

It is fervently to be wifhed, that the plenipotentiaries may prevail upon the potentates they reprefent, whether monarchical or republican, to declare war againft this formidable enemy to the human race, the fmall-pox, which, it is confidently afferted by Dr. Fauft deftroys, in Germany alone, *feventy thoufand perfons annually, or nearly* 200 *per day !*

---

Dr. JOHN ARCHER, of Harford county, Maryland, in America, has found by a number of decifive experiments recently inftituted, that the fenekaroot (*Polygala Senega, Lin.*) is an almoft infallible remedy in Croup, or the *Cynanche Trachealis* of *Cullen*. From a letter lately written by Dr. Archer, and addreffed to Dr. Barton, of Pennfylvania College, we fhall communicate to our readers the following important particulars:

" I have (fays Dr. Archer) in a great many inftances found a decoction of the *Seneka* the moft powerful medicine in the cure of this difeafe, and I am happy to tell you that I believe it may be depended on. I make a ftrong decoction of the root in the following manner, viz. half an ounce of the feneka, in coarfe powder is boiled in eight ounces of water, down to four. Of this I give a tea-fpoonful every half hour, or hour, as the ur-

* The plan itfelf fhall be given in a future number.

urgency of the fymptoms may require, and at intervals a few drops, to keep up the ftimulus, until it either acts as an emetic or cathartic. I then repeat it, in fmaller quantities, fo as to preferve the ftimulus of the feneka conftantly in the mouth and throat.

"If the difeafe be more advanced, and the breathing more difficult, with a peculiar harfh or fhrill found, like air forcibly drawn through a fmall aperture: attended with a retraction of the upper part of the abdomen under the cartilages of the ribs; I then give calomel freely and frequently, and rub mercurial ointment on the throat and contiguous parts, fo as to affect the glands of the throat and mouth, as quickly as poffible. This I do that the mercury may co-operate with the action or ftimulus of the feneka, and thereby haften the feparation of the membranous fubftance formed in the trachea.

"In this method I have fucceeded in the cure of the croup, even beyond my moft fanguine expectations."

---

Dr. Struve, of Gorlitz (who was lately prefented by the Royal Humane Society of London with all their works, for his excellent treatifes on re-animation, and other fubjects connected with the philanthropic views of the fociety) has difcovered a remedy for the *hooping cough*, which is intitled to the ferious attention of practitioners.—After having directed a gentle emetic to be taken by the patient, Dr. Struve prefcribed the following mixture to be rubbed in, every two hours, in fmall quantities, about the region of the ftomach. One fcruple of emetic tartar is diffolved in two ounces of water, to which folution is added one ounce of the ftrong tincture of cantharides*.—In a great variety of inftances, the doctor obferved, that a gentle perfpiration came on during the night, after the ufe of this application; that the violence of the cough immediately abated, and in a fhort time the fymptoms totally difappeared. He does not confider the emetics alone adequate to produce thefe beneficial effects; but, in combination with the mixture for external application, the difeafe feems no longer fo formidable as it has hitherto been. We cannot, however, forbear, to caution the young practitioner in the ufe of this, and fimilar heroic remedies, particularly if reforted to, from urgent neceffity, in infantile and female diforders.

---

Profeffor Le Roy, of the fchool of medicine at Paris, has lately publifhed an account of a new method of curing eryfipelatous inflammations, and cutaneous eruptions, by the external application of *hot flower;* detailing feveral cafes in which that remedy has been fuccefsfully ufed. For want of room in the prefent number, we propofe to give a more particular account of this interefting difcovery in our next.

---

According to an account given by Dr. De Witt, phyfician in the city of Albany, the effects of the *datura ftramonium*, thorn-apple, appear to be fo extraordinary, that they well deferve the further notice and inveftigation of practitioners. The feeds of this plant, when eaten or fwallowed inadvertently, have been found to produce, 1. unufual pain and anxiety; 2. convulfive motions; 3. apparent dread or averfion to water or fluids of any kind; 4. vefications on the fkin, after the violent fymptoms had fubfided; and 5. large and involuntary difcharges of urine. Thus, the extremely active and ftimulating power of this plant, and its violent operation on the human body, would feem almoft to equal the combined force of plague, fmall-pox, hydrophobia, and epileptic convulfions.

From

* We have no doubt that this composition is, *in substance*, the same with Roche's Royal Embrocation for the hooping cough.

From a letter of Citizen S. lately published in the "*Annales de Chimie*," it appears that no less than twenty-seven of his poultry, including a turkey-hen, all died in the course of a few days, in the most dreadful convulsions. Curiosity induced him to open them, when every thing appeared in a sound state, without any indications of the slightest malady; he perceived, however, that the internal membrane of the gizzard was somewhat tough and shrivelled, like most animal substances, when exposed to the action of heat. In all the different subjects the stomachs were luminous; the grains not fully digested, glittered in falling down to the ground; and those which at first presented no light, almost instantly exhibited both light and the smell of phosphorus, when heated. This convinced Citizen S. that there could be only one cause for all these effects, and that they were all produced by the circumstance of his having, four or five days before, thrown out some water through a casement into the poultry yard; which water had served to wash and purify several substances, on which operations of phosphorus had been performed. The phosphorus contained in these waters, in a state of nature, he considered as solely occasioning the death of such a number of domestic animals.

---

The *Chemical Society of Philadelphia* have appointed a committee to collect, into *one synoptical view*, all the different processes carried on in different countries, for the purpose of manufacturing *nitre*. They invite all persons who may possess information respecting the mode of producing this valuable *neutral salt*, to forward it to their secretary *(post paid)*. They also request their fellow-countrymen to furnish them with accurate descriptions of the *situation*, *soil*, *temperature*, &c. &c. of those places in which nitre is found in a *native state*.

---

The liquid obtained from distilled animal substances, and which has hitherto been supposed to contain only carbonate of ammonia, and an oil, has been found by recent experiments made by Citizen Berthollet, to contain also an acid, to which he has given the name of *zoonic acid*. This acid, which he has discovered in the liquid produced from the gluten of meal, from the yeast of beer, from bones and rags distilled for the preparation of the muriate of ammonia, he thinks himself authorized to consider as a product of the distillation of all animal substances. To obtain the zoonic acid pure, he employs the following process: He mixes water of the zoonate of lime, well assimilated in a tubulated retort with phosphoric acid, and then proceeds to distillation. The zoonic acid is somewhat volatilised; it requires a degree of heat nearly equal to that of boiling water to arrive at distillation; it will then be necessary to boil the liquor; if two receivers be used together, none will pass into the second. It appears that a part of the acid is destroyed by the action of heat; for the liquid upon boiling becomes brown, and at the close of the operation grows black. Hence Citizen Berthollet infers, that this acid contains carbone. He has not yet ascertained the other principles which become disengaged in the decomposition. The zoonic acid has a smell, not unlike that of meat when roasted brown; and in effect, it is produced on that occasion. Its taste is pungent (austere) It gives a strong red to paper died with the turnsole, and effervesces with all alkaline carbonates, &c.

---

Citizen Brugnatelli, author and editor of the "*Italian Annals of Chemistry*," has lately declared his concurrence with the opinion expressed by Citizens Fourcroy and Vauquelin, relative to the experiments of Goettling, in Germany, on the pretended combustion of phosphorus in azotic gas.

gas. Brugnatelli here expreſſes his aſſent to the evidence afforded by the experiments of the French chemiſts, and he accordingly retracts the denomination of *foſſigene gas* (engendering light) which formerly he thought himſelf intitled to give to azotic gas, on the facts announced by the chemiſt of Jena;—the denomination of *thermoxigene gas* (engendering heat and acids) muſt conſequently fall, at the ſame time, with that of foſſigene.

---

Mr. Collier's Patent "*for a chemical proceſs for freeing fiſh-oils from their impurities in ſmell, taſte, and colour; and for improved ſtrainers for oil, water, and other liquids, with inſtruments for aſcertaining their qualities, and aſſiſting their burning*," is of ſuch extenſive *practical* importance to health, domeſtic economy, and the arts, that juſtice requires this very ingenious and uſeful invention ſhould be recorded in the annals of medical and phyſical ſcience—The excellent machines contrived by this philoſophical artiſt, are extremely ſimple in their conſtruction, and particularly his improved *ſtrainers* or *filtering machines* for water and other fluids, poſſeſs the very important advantage, that they may be readily adapted to the purpoſes of families, work-houſes, hoſpitals, public charities, the navy, or the merchant ſervice, and alſo to the more particular uſes of oilmen, of diſtillers, of the laboratory, the brewery, &c. A prominent feature in theſe inventions, is the manner of diſtilling, without heat; which, if applicable to the purpoſes ſet forth in the ſpecification of the patent, (See "*Monthly Magazine* for January") may form a new epoch in the hiſtory of chemiſtry. The deſign of this contrivance is, to free all fluids from their colour, and putreſcent qualities, almoſt without any expence of fuel. In a word, we are perſuaded that the king's patent has never been more deſervedly granted, than in the preſent inſtance; as the generality of patents, obtained for *medicinal purpoſes*, have too often reflected diſgrace on the royal ſignature, by giving an authoritative ſort of ſanction to the fanciful productions of mercenary empirics, and ignorant dabblers in medicine. How long will theſe nuiſances of ſociety be permitted to carry on their hazardous experiments and nefarious practices?

---

An aſſociation has been formed in the city of New York, for *the inveſtigation of the mineral and foſſil bodies* which compoſe the fabric of the globe, and more eſpecially for aſcertaining the natural and chemical hiſtory of the minerals and foſſils of the United States, under the name of *The American Mineralogical Society*.—Specimens of ores, metals, coals, ſpars, gypſums, cryſtals, petrifactions, ſtones, earths, ſlates, clays, chalks, lime-ſtones, marbles, and every foſſil ſubſtance that chance or time may diſcover, or which may fall in the way of a traveller, and tend to illuſtrate the mineralogical hiſtory of America, will be examined and analyzed *free of charge*; ſufficient pieces, with the owner's leave, being reſerved to be placed in the ſociety's collection.

---

The committee of the *American Mineralogical Society* have lately publiſhed an advertiſement, the object of which is, to concentrate in one compendious view, all the information that now lies ſcattered through the different States of the Union, relative to the means America poſſeſſes of furniſhing objects *immediately* requiſite for *national defence*.

---

Dr. Benjamin De Witt, of Albany, whoſe account of the deleterious effects of *Stramonium* we have before noticed, intends ſpeedily to publiſh a deſcriptive account of all the principal *mineral and medicinal waters*, found in the State of New York. Few places abound with ſo many and ſuch valuable mineral mineral waters as the northern parts of this State ; and as no general hiftory has hitherto been given of their compofition, or of the ufeful purpofes they are calculated to ferve, a work of this nature may be juftly accounted *an American defideratum.*

---

A *mineral fpring* has been lately difcovered in *Clinton* county, ftate of *New York*, containing very large quantities of a faline fubftance, which, from fome flight trials, is fuppofed to confift chiefly of Epfom falts, or fulphat of Magnefia.

---

The *American Philofophical Society* have lately publifhed a circular letter, in which, among other objects of inquiry, relative to the antiquities, cuftoms, manners, languages, and character of the Indian nations, they invite their countrymen to inftitute refearches into the natural hiftory of the earth, the changes which its furface has undergone as to mountains, lakes, rivers, meadows, &c.

---

Mr. THOMAS BRUFE, Sen. of Cheftertown, Maryland, advertifes patent inftruments for *extracting teeth in a perpendicular direction.* His advertifement is accompanied with certificates, in favour of his inftruments, from Drs. SHIPPEN, WILKINS, and GOODWIN. From the well-known talents of thefe gentlemen, there is reafon to expect much advantage from this invention, in the operation of tooth-drawing.

---

The ftudent of *Mineralogy* will learn with fatisfaction, that he may be fupplied from Leipzig with cabinets, containing fpecimens of minerals, arranged in fyftematic order, and at the following moderate prices.

A collection of 150 fpecimens, each depofited in a feparate pafte-board cafe, and numbered, correfponding to a catalogue, in which a defcription is given of every piece, at 6 rix dollars Saxon currency, or about £ 1. 1. 0. Britifh :—a more extenfive collection confifting of 250 fpecimens of fuperior value, furnifhed with a fimilar catalogue, and packed in drawers, in a red painted cafe, with lock, &c. for twenty rix-dollars S. C. or about 3l. 6s. 8d. and laftly, collections of the beft fpecimens, amounting to 500 in number, the produce of various countries of Europe, many of which are rare, and as yet non-defcript, being recently collected, and provided with a complete and inftructive catalogue, &c. &c. for 60 dollars, or about ten guineas.

Amateurs are requefted to addrefs themfelves to Mr. Martini, bookfeller, in Leipzig.

---

According to the half-yearly *catalogue of lectures* delivered during the prefent feafon in the *Univerfity of Jena*, we find forty feparate courfes announced on the different branches of medicine, befides thofe given on clinical cafes. Among this great diverfity of fubjects we fhall gratify the Englifh reader by mentioning the following, which will doubtlefs appear rather extraordinary. On medical anthropology, Profeffor LODER ; on the opinions refpecting the vital principle, Dr. ECKHARD ; on fome principles of the Brunonian theory, Profeffor STARCK ; on popular or domeftic medicine, Dr. BRETSCHNEIDER ; on the art of writing prefcriptions, NICHOLAI and GRUNER ; on the advantages of difputations and examinations, FUCHS, BRETSCHNEIDER, and BERNSTEIN.—We fhall, upon this occafion, only apply the remark of SENECA : *Confufum eft quidquid in pulverem fectum eft.*

At

At a late promotion of a candidate, Mr. Diruf, to the dignity of doctor of Phyfic, in the German univerfity at Heidelberg—a folemnity which the reigning Duke and Duchefs of Deux-Ponts honoured with their prefence, the privy-counfellor, Dr. Mai, delivered a latin oration on the following curious fubject: "*Quænam ex paradoxa Brunonis doctrina in praxin medicam emolumenta?*" This fingular queftion was not the only one here propofed, but the learned candidate gave a counterpart to it, by another oration in which he anfwered a ftill more extraordinary query, viz. "*Quænam ex Brunonis doctrina fata pertimefcenda?*"

---

The *Medical Lectures* in Columbia college, New York, commence annually on the fecond Monday of November, on the following branches:

*Chemiftry and Natural Hiftory;* by Profeffor Mitchill.
*Anatomy and Surgery* ——— Post.
*Midwifery, and Infantile Difeafes* ——— Rodgers.
*Theory and Practice of Phyfic* ——— Hammersley.
*Materia Medica and Botany* ——— Hosack.

Regular *Clinical Lectures* are alfo given by Dr. Rodgers, in the New York hofpital; which is furnifhed with a valuable collection of medical books.

---

At the meeting of the *College of Phyficians of Philadelphia*, held July 3d, 1798, the following officers were elected, according to the ufual forms: Dr. John Redman, *Prefident;* William Skippen, Jun. *Vice-Prefident;* Adam Kuhn, Samuel Duffiled, Thomas Park, and Caspar Wistar, *Cenfors;* Benjamin Say, *Treafurer;* and Thomas C. James, *Secretary.*

---

The newly-eftablifhed colleges in the *weftern hemifphere* have fet a laudable example, in directing their medical ftudents, to write the *Inaugural Differtations* for the degree of Doctor of Phyfic, in their native tongue, or more properly, in the language tranfplanted from Britain to America.—We are happy to obferve, that this fpecimen of good fenfe has lately been introduced likewife in fome of the *German* colleges. The firft of thefe, which broke through the abfurd cuftom of writing them in Latin, was the univerfity of Kiel, in the principality of Holftein, fubject to the king of Denmark.—Mr. William George Pfefferkorn, now Phyfician to the Infirmary at Altona, was the firft graduate in Germany, on whom the titles of Doctor of Medicine and Surgery were thus conferred, after having written his differtation "*On the difeafe of the Norwegians, called* Radefyge," publifhed at Altona, (in German) 1797.

---

Among the numerous tranflations of Englifh books into the German language, whether of a valuable or frivolous complexion, we fhall, at prefent point out the following, beginning with thofe which we confider to poffefs the moft merit: "Dr. G. Fordyce's *Practice of Phyfic;*" from the fixth edition; by Dr. C. F. Michaelis: 270 pp. 8vo.—"Dr. R. Willan's *Defcription and Treatment of cutaneous Difeafes,*" Ord. I. 110 pp. quarto.—"Dr. J. Currie's *Medical Reports on the Effect of Water, cold and warm, as a Remedy in Fevers, &c.*"—"A. Thomson's *Inquiry into the Nature, Caufes, and Cure of nervous Diforders;*"* tranflated from the fourth Englifh edition,

* The German Reviewers exprefs an uncommon and uniform fatisfaction at the excellent rules of diet contained in this fmall treatife; as, without due attention to fuch rules, it is no eafy task to cure a hypochondriac. W.

edition, and accompanied with remarks by Dr. G. F. Muhry, 83 pp. 8vo.—" Dr. W. Falconer's *Observations on the Pulse, &c.*" with remarks and a supplement, by Dr. Kausch, 178 pp. 8vo.—" Dr. Bree's *Practical Inquiry on disordered Respiration, &c.*; with remarks, by K. F. A. S. (to whom Dr. Michaelis, of *Leipzig*, who first announced a translation of this work in the Jena Gazette, has consigned the task.)—" Dr. Crichton's *Inquiry into the Nature and Origin of mental Derangement, &c.*" 2 vols. 8vo. Goettingen, 1798—" Dr. T. Trotter's *Medicina Nautica*; translated by Dr. C. Warner, and accompanied with a learned preface, by Professor Hufeland.—" T. Blizard's *Suggestions for the Improvement of Hospitals, &c.*"

---

The indefatigable Dr. Beddoes of Clifton is preparing for the press the first volume of a periodical publication, intitled " *Contributions to Physical and Medical Knowledge: principally from the west of England and Wales;*" the profits of which (if any accrue) are to be devoted to the *Institution for investigating the medicinal powers of factitious airs.*—In our next number we hope to be enabled to give an account of the progress of this Institution; and for the last publication of Dr. Beddoes, which is a valuable " *Collection of Testimonies, respecting the treatment of the venereal disease, by nitrous acid,*" we refer the reader to our *Retrospect of Medical Literature;* in the concluding part of this Journal.

---

Dr. Underwood's " *Treatise on the Diseases of Children, now first adapted exclusively to the use of Professional Readers,*" is in the press, in an advanced state, and nearly ready for publication. With the same work, the Doctor proposes to publish his " *Chirurgical Tracts.*"

---

Mr. Charles Brown, of Ely-Place, Holborn proposes to publish, as early as possible, a volume, intitled: *Annals of Pneumatic Medicine,*" for the completion of which he solicits the assistance of such gentlemen as are in possession of any facts, relative to the use and effects of factitious airs in diseases.

---

The third edition of Dr. Thornton's " *Medical Extracts*" with a number of additional plates and many considerable improvements, is now in the press and will speedily be published.

---

The second edition of I. Townsend's " *Physicians, Vade mecum,*" or an abridged translation of Dr. Cullen's System of Nosology, &c. a very useful manual to the medical student, will appear in a few weeks.

---

The learned Greek, Coray, has now ready for the press an elegant version, in German, of the celebrated work of *Hippocrates, on air, water, and climates.* This translation is highly recommended by Professor Sprengel, and will appear at Leipzig in the course of next summer. Of this inestimable work we have authority to announce a speedy translation into English, by Dr. W. Wright.

---

A work of considerable interest and importance to the medical world, is now in the press at Gotha, intitled: *The History of Inventions, Theories, and Systems, which have prevailed in Physics and Medicine, during the Eighteenth Century.* This historical retrospect is intended to supply the readers of the

 reputed reputed *Journal of Inventions, &c.* with all the facts and diſquiſitions contained in the firſt twenty-four numbers of that *excellent* periodical publication, now out of print. As we have a copy of this Journal in our poſſeſſion, we can aſſert, with a degree of confidence, that its leading principles are founded in rigid impartiality and juſtice, though occaſionally we find ſome parts tainted with a ſmall portion of egotiſm. The very learned, but acrimonious Profeſſor HECKER, is the ſuppoſed Editor of this hiſtorical Journal, in which the modern theories of *oxygenating the ſyſtem—of the chemical action of the gaſes on, or within, the human body,* &c. &c. are particularly and very keenly conteſted.

---

An abridgment, in two volumes octavo, of that complete and valuable work, *The Phyſical Dictionary,* by the late Doctor and Profeſſor GEHLER, (in ſix volumes octavo, with a great number of plates) is now in the preſs at Leipzig; it is prinicipally calculated for the uſe of ſuch ſtudents of Natural Philoſophy, as may not find it convenient to purchaſe the larger and more expenſive work.

---

Mr. ACCUM, an ingenious foreigner, reſident in London, propoſes to publiſh (by ſubſcription) a work intitled, *Chemico-Phyſical Aphoriſms, comprehending a general View of the Diſcoveries, Experiments, Obſervations, Phenomena, and Facts illuſtrated in Experimental Chemiſtry; with a ſummary Deſcription of the Uſe and Management of the principal Apparatus employed by the Moderns in Natural Philoſophy.*

---

A faithful tranſlation of a late work of conſiderable merit, and containing much novel matter, has juſt come from the preſs, but too late to give a particular account of it, in our "*Retroſpect of Medical and Phyſical Literature.*" —It is intitled: "*Travels in England, Scotland, and the Hebrides, undertaken for the purpoſe of examining the State of the Arts, the Sciences, Natural Hiſtory, and Manners of Great Britain: containing mineralogical Deſcriptions of the Country round Newcaſtle, of the Mountains of Derbyſhire, &c. &c.*" Tranſlated from the French of B. FAUJAS SAINT-FOND, member of the National Inſtitute, and profeſſor of geology in the muſeum of Natural Hiſtory at Paris. *London.* Ridgway.

---

A new and enlarged edition of Dr. J. C. SMYTH's work on the jail-diſtemper, and particularly on the extirpation of contagion, by means of fumigating (with nitrous gas) rooms and places filled with peſtilential vapours, is nearly ready for publication, and will appear in the preſent month.

---

Dr. E. G. CLARKE's ſmall work, intitled "*Medicinæ Praxeos Compendium,*" which has been announced ſome time ago, will certainly appear early in this month.

---

Mr. A. CARLISLE, we underſtand, will not be able to deliver his uſual courſe of lectures on the general ſcience of anatomy, in the preſent ſeaſon; on account of other engagements.

